# Parental well-being in times of Covid-19 in Germany

**DOI:** 10.1007/s11150-020-09529-4

**Published:** 2021-01-14

**Authors:** Mathias Huebener, Sevrin Waights, C. Katharina Spiess, Nico A. Siegel, Gert G. Wagner

**Affiliations:** 1 DIW Berlin, Berlin, Germany; 2 IZA Bonn, Bonn, Germany; 3 CEP at the LSE, London, UK; 4 Freie Universität Berlin, Berlin, Germany; 5 Infratest dimap, Berlin, Germany; 6 Max Planck Institute for Human Development, Berlin, Germany; 7 CESifo, Munich, Germany

**Keywords:** Well-being, Covid-19, Corona virus, Family, Children, Day care closures, School closures, COMPASS, SOEP, D1, H12, H75, I2

## Abstract

We examine the effects of Covid-19 and related restrictions on individuals with dependent children in Germany. We specifically focus on the role of day care center and school closures, which may be regarded as a “disruptive exogenous shock” to family life. We make use of a novel representative survey of parental well-being collected in May and June 2020 in Germany, when schools and day care centers were closed but while other measures had been relaxed and new infections were low. In our descriptive analysis, we compare well-being during this period with a pre-crisis period for different groups. In a difference-in-differences design, we compare the change for individuals with children to the change for individuals without children, accounting for unrelated trends as well as potential survey mode and context effects. We find that the crisis lowered the relative well-being of individuals with children, especially for individuals with young children, for women, and for persons with lower secondary schooling qualifications. Our results suggest that public policy measures taken to contain Covid-19 can have large effects on family well-being, with implications for child development and parental labor market outcomes.

## Introduction

Life has changed dramatically for individuals in many countries as a result of the spread of Covid-19 and the implementation of measures to control the pandemic. Such far-reaching crisis-induced policy regulation has rarely been seen in democratic nations since the end of World War II. Some of the restrictions, such as nationwide closures of schools and day care centers, have had particularly strong impacts on parents of dependent children. While closures of schools and day care centers have led many parents to spend more time with their children, the measures have also resulted in a fairly sudden breakdown of established routines for combining work life, family life, and other activities. Many parents have cut their working hours (and, hence, family income) or have attempted the difficult task of combining working from home (if at all possible) with looking after children (e.g., Andrew et al. 2020; Del Boca et al. 2020) or find alternative childcare. Other factors have also had a particular impact on families, including bans on social contact, shutdowns of economic activity, and fear of the pandemic. In general, how the crisis and its unique, manifold, impact on family settings have affected parents is subject to broad public and growing academic debate.

In this study we ask the question: what is the differential effect of the Covid-19 crisis on parents of dependent children over other individuals? Put another way, we aim to estimate the effects on parental well-being of the particular aspects of the Covid-19 crisis that affect parents only, such as day care and school closures. Other studies focus on general declines in well-being as a result of the fear of the virus and negative economic impacts (Lu et al. 2020; Béland et al. 2020b; Cheng et al. 2020; Fetzer et al. 2020a; Fetzer et al. 2020b) or loneliness as a consequence of physical distance during lockdown regimes (Armbruster and Klotzbücher 2020; Brodeur et al. 2020; Brülhart and Lalive 2020; Hamermesh 2020; Knipe et al. 2020; Tubadji et al. 2020). However, very few studies look at the specific impact on parents or investigate the role of additional childcare responsibilities (two exceptions, discussed below, are Adams-Prassl et al. 2020b and Etheridge and Spantig 2020). Parental well-being is an important outcome, both in itself and as a predictor of negative child outcomes (e.g., Berger and Spiess 2011; Camehl et al. 2020; Smith 2004; Mensah and Kiernan 2010; Spinelli et al. 2020, Griffith 2020; UKE Hamburg 2020), relationship dissolution or divorce (Frank and Gertler 1991), costs to the economy (e.g., Oswald et al. 2015; Naylor et al. 2012; McDaid 2011), and even compliance with measures introduced to stop the spread of Covid-19 (Krekel et al. 2020). As such, policymakers may wish to know the magnitude of impacts on parental well-being in order to decide on optimal lockdown policies and to direct remedial policy, such as mental health interventions during the pandemic and in its aftermath.

Our first contribution is an up-to-date analysis of a unique collection of data on parental well-being. We use a new data set to document the evolution of well-being during the Covid-19 pandemic for individuals with and without dependent children in households in Germany. The COMPASS study conducted by “infratest dimap” is based on a representative sample of the German population eligible to vote in Germany and with an online access.^1^ As such, it is one of the few representative surveys of well-being that exists for Germany, and the largest well-being survey taken during the Covid-19 crisis.^2^ Our main analysis is based on 14,781 observations of 8977 individuals reporting on their well-being in May and June 2020. The data includes satisfaction in three areas that are important for the well-being of families, namely general life satisfaction, satisfaction with family life, and satisfaction with childcare. The data also includes detailed questions on whether individuals with dependent children in the household are affected by day care and school closures, on the degree to which they feel restricted by public measures taken to contain Covid-19, and on the extent to which they work from home.

Our second contribution is a descriptive analysis of how well-being under Covid-19 compares with well-being in a pre-Covid-19 period for various subgroups. We make use of the COMPASS survey to describe well-being during Covid-19 and the German Socio-Economic Panel (SOEP) to describe well-being in the pre-Covid-19 period (2018). The SOEP is a representative survey of households that includes the same questions on well-being that are asked in the COMPASS study. Motivated by a literature that predicts heterogeneous impacts of the crisis and differential reliance on publicly-funded childcare by gender and socioeconomic status (e.g., Alon et al. 2020; Conti 2020; Jessen et al. 2020), we examine how the change in well-being between the two surveys varies by age of the youngest child, by parental gender, and by parental education. We make these comparisons for our sample period when schools and day care centers were largely closed but when many other restrictions had already eased and new infection rates were pervasively low. As such, we assume the schools and day care center closures, or at least the restricted access to permanent schooling and day care, are a major factor in the differences of well-being, especially for families with young children.

Our third contribution is to supplement the descriptive analysis with estimates of the effect of the crisis on individuals with children relative to individuals without children using a difference-in-differences (DiD) design. Goodman-Bacon and Marcus (2020) argue that DiD designs are well fitted for evaluating the effects of Covid-19. The comparison with an unaffected group accounts for changes in well-being that might be unrelated to the crisis, e.g., due to overall time trends or to the use of different survey methods, as well as the general shift in well-being due to the crisis (i.e., for reasons not particular to parents). The resulting DiD estimate captures changes in well-being resulting from factors that affect parents only, principally the closures of schools and day care centers. The validity of the DiD relies on a parallel trends assumption: that the well-being of individuals with children would have followed a similar path to the well-being of individuals without children in the absence of the crisis. We provide evidence on parallel trends in the pre-period.

Our DiD estimates find significant declines in satisfaction for individuals with children relative to individuals without dependent children. The negative effects are larger for parents of younger children, for women, and for those with lower educational attainment and are larger for parents that report being affected by closures of day care centers and schools. The results are robust to several sensitivity checks that significantly adjust our samples and definition of the outcome variables. Our findings are consistent with Etheridge and Spantig (2020), who find reduced well-being during the pandemic that is greater for parents with childcare responsibilities in the UK. However, our results contrast with Adams-Prassl et al. (2020b) who find that declines in well-being are not related to additional childcare responsibilities. This difference perhaps arises because they focus on a period characterized by a general stay-at-home order in the US whereas we look at a period when the lockdown is eased but schools and day care centers are still mostly closed. Another reason could be the different childcare systems in each context. Ours is the only study we are aware of to examine impacts on several important dimensions of well-being by age of the children in the household.

Our findings contribute to a literature that documents the uneven impacts of the Covid-19 crisis by gender and socioeconomic groups across many dimensions. Studies from several countries find that women have larger declines in well-being than men during the crisis (Adams-Prassl et al. 2020b; Davillas and Jones 2020; De Pedraza et al. 2020; Etheridge and Spantig 2020). Looking at other outcomes, Del Boca et al. (2020) and Andrew et al. (2020) find that women bore the majority of the additional workload (childcare and housework) in Italy and the UK, Adams-Prassl et al. (2020a) find women are more likely to lose a job, and Béland et al. (2020a, 2020b) highlight increased domestic violence as an outcome of family stress. Furthermore, research suggests that children of lower educational backgrounds have worse learning conditions at home (Huebener and Schmitz 2020) and will lose the most from school closures in terms of educational achievement (Eyles et al. (2020). Thus, our findings on parental well-being are consistent with the literature that finds the crisis affects women and those from lower educational backgrounds disproportionately.

Our standardized estimates indicate that life satisfaction declines by between 0.13 and 0.28 standard deviations (depending on age of the youngest child) relative to individuals without children. Impacts on satisfaction with family life and on satisfaction with childcare are larger still. In comparison, Etheridge and Spantig (2020) find declines in well-being in the UK for individuals who have not lost their job to be 0.26 standard deviations for women and 0.13 for men, implying an average overall decline that falls within our range of estimates. Adams-Prassl et al. (2020b) find that stay-at-home orders result in declines in mental health of around 0.09 standard deviations in the US. Thus, our differential effect for parents (i.e., the decline for parents over non-parents) in Germany is similar in size or larger to the total effect for all individuals in the US and the UK. This large estimated effect may reflect the major reliance on publicly-funded, universal day care by almost all families in Germany. Our effects also compare to estimates on the impact of provision of publicly-funded day care on maternal well-being. For example, Schmitz (2020) finds that the general life satisfaction of mothers increases by 0.30 standard deviations if their child attends day care due to increased provision.

## Institutional and Covid-19 policy background in Germany

**Fig. 1:**
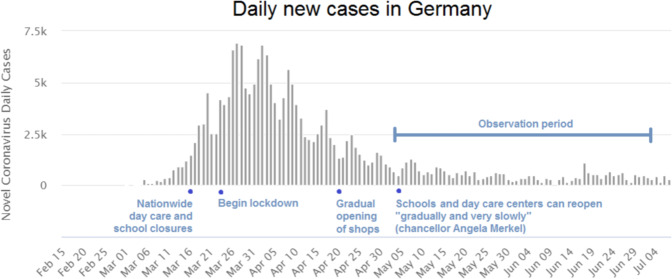
Number of daily coronavirus cases in Germany and data availability on individuals’ satisfaction levels. *Notes:* The figures shows the course of new infections with Covid-19 in Germany 2020, as well as selected dates for political decisions in the course of the pandemic. It also shows the period for which this report analyses data on satisfaction. *Source:* Own illustration based on WHO, John Hopkins University (2020): Development of the daily reported number of new cases of coronavirus (COVID-19) in Germany since January 2020 (as of 8 July 2020) (accessed on 8 July 2020 from https://www.worldometers.info/coronavirus/country/germany/)

To curb the spread of Covid-19 in Germany, almost all schools and day care centers were closed from March 16 onward (see Fig. 1), with emergency day care being available only to families in systemically relevant occupations. For most families, central care and educational opportunities for their children were no longer available. In April, the German National Academy of Sciences, Leopoldina, released a statement suggested that day care centers and schools should be kept closed until the summer holidays (Leopoldina 2020). This statement was the focus on significant attention and was discussed controversially in the public.^3^ At the same time, politicians advised against having grandparents provide childcare due to the increased health risk for older people and the great danger of infection with Covid-19. Since May, the scope of childcare offer by day care centers and schools in the various federal states has gradually expanded. However, a return to regular operations was not scheduled in most of the 16 federal states until after the summer holidays. Even then, important questions remain regarding how regular schooling and care will be organized under exceptional hygiene measures. The focus of our analysis is on the period covered by the months of May and June 2020, when schools and day care centers were still closed to most children, but by which point the shutdown of activity and restrictions on social contact often referred to as ‘lock-down’ (as of March 23) was largely relaxed. In May, about 79 percent of the respondents with children under six stated that they were affected by day care center closures, falling to 75 percent in June. The proportion of respondents affected by school closures was 89 percent in May and 83 percent in June.^4^

Day care and school closures are particularly constraining to family life because several policy measures since the turn of the century have promoted a substantial increase in maternal labor supply in Germany. These measures include the increased supply of publicly funded day care (e.g., Spiess and Wagner 2003; Spiess 2008; Bauernschuster and Schlotter 2015; Müller and Wrohlich 2020). Since 2000, enrollment has been almost universal for children aged three years and older. Below age three, the proportion of children in day care is at about 34.3 percent in 2019, with considerable variation across regions (Autorengruppe Bildungsberichterstattung 2020). Moreover, the number of children aged three or older in full-time day has also increased: in 2019 about 52 percent of all children 3 years and over attended day care 35 h per week or more (Autorengruppe Bildungsberichterstattung 2020). Next to the expansions in the availability of care, several states also reduced or removed parental contributions to day care, which evidence suggests may have increased mothers’ working hours for (Huebener et al. 2020).^5^ For school aged children, a large federal policy initiative starting in 2004 promoted the expansion of all-day schooling for primary school-aged children, also promoting maternal employment (e.g., Gambaro et al. 2018). In 2019, 50 percent of all children in primary schools either attend an all-day school program or attend an after-school care-club (*Hort*, Autorengruppe Bildungsberichterstattung 2020). Based on these policy measures, maternal labor force participation in Germany rose faster than the European average (OECD 2019). In 2015, around 63 percent of mothers whose youngest child was aged between three and five were part of the labor force; of these, 30 percent worked full-time. Paternal labor supply is consistently very high, with most fathers working full-time (see, e.g., Huebener et al. 2020).

## Data and methods

### Data source 1: COMPASS survey data

Our analysis is based on exclusively collected data from the COMPASS survey carried out by the private research institute “infratest dimap”.^6^ The survey aims at closely tracking current developments in the German population during Covid-19, with a particular focus on agreements/disagreements with policy measures taken to contain Covid-19, and to measuring the extent to which restrictions affect individuals. For this purpose, between 250 and 350 persons have been surveyed each day since March 12. The survey records basic demographic characteristics, the household structure, the age of children in the household, general values and attitudes, as well as personal traits.

The COMPASS survey is carried out on the basis of a random sample, within an online access panel, the “Payback Panel”. This panel is recruited on the basis of membership in Payback, Germany’s largest consumer bonus program, consisting of approximately 25 million consumers, covering about every second German household. In contrast to many other access panels available for online research, participants in the Payback panel were recruited offline and were unable to self-recruit, limiting problems arising from self-selection. For the COMPASS survey, infratest dimap used more than 80,000 panelists to draw same-day samples with respect to age, gender, education, and federal state. In order to minimize sample distortions, the survey data were weighted in such a way that the composition of the samples in terms of gender, age, schooling, and region (East/West) corresponds to the composition of the Federal Statistical Office’s Micro Census from 2018. The results claim to be representative, by weighting, for those eligible to vote in Germany with online access. Based on statistics of the German Federal Statistical Office from 2019, 90 percent of the German population uses the internet daily, and another 8 percent at least once a week. In the 16–44 age group, which is most relevant for the analysis of parental well-being of parents of young children, the proportion of daily users is over 98 percent (Destatis 2020).

Our main analyses is based on 14,781 interviews conducted between May 1 and July 1, 2020, comprising 8977 people, of whom 5804 were interviewed twice.^7^ We use satisfaction with life in general, satisfaction with family life, and with satisfaction with childcare as our main outcome variables. Respondents rate their own satisfaction in the various areas on an 11-point Likert scale ranging from 0 (not satisfied at all) to 10 (very satisfied; see Headey et al. 2010). Additionally, we use the information on whether respondents were affected by day care center and school closures and whether they were asked by their employer to work from home. We also evaluate whether respondents feel restricted in their everyday life by Covid-19 and related policy measures. We define ‘individuals with dependent children’ as those living in the same household as a child younger than 16 years. We define individuals without dependent children as those that do not live together with a child or where the youngest child in the household is 16 or older. For simplicity, in this paper we often only refer to the former group as ‘parents’.^8^

### Data source 2: German Socio-Economic Panel (SOEP)

We support our main analysis with data from the German Socio-Economic Panel Study (SOEP, see Goebel et al. 2018). As of 2018, this annual representative household panel study interviews about 33,000 individuals in 11,000 households on a broad range of topics, including the same questions on general life satisfaction, satisfaction with family life, and satisfaction with childcare that are used in the COMPASS survey. The SOEP survey is typically conducted in face-to-face interviews. We use the most recent survey wave that is available for the scientific community, conducted in 2018 (SOEP v35), to characterize well-being in the population in the period preceding Covid-19.

Our SOEP sub-sample includes all persons aged 18 or older who are eligible to vote in Germany and who answered questions on life satisfaction, family life, and childcare in 2018. SOEP also includes very old people in the data set. For even better comparability, SOEP respondents over 70 years of age were excluded from the sample. Thus, the target population of the analyses is largely identical to that of the COMPASS dataset. The results were weighted with the individual weighting factor in order to be representative of the underlying population.

Table 1 presents descriptive statistics on both samples. The average age is 45.6 years in the SOEP, and 45.4 in the COMPASS data, with very similar age distributions across both datasets. The share of observations with upper secondary schooling is 38 percent in both datasets. The share of households with no children below age 16 is 0.77 and 0.74 in the SOEP and COMPASS data, respectively. Descriptive statistics on the unweighted samples are reported in Appendix Table 5. They reveal that socio-economic and socio-demographic characteristics are not equally distributed in both surveys. For example, during Covid-19, households without dependent children are underrepresented, while older individuals and individuals working in white-collar professions are overrepresented.^9^

**Table 1:** Descriptive statistics

	2018 (SOEP v35)		2020 (COMPASS)	
	Mean	(SD)	Mean	(SD)
*Individual characteristics*				
Female	0.49	(0.50)	0.51	(0.50)
Age in years	45.59	(14.85)	45.44	(14.14)
Below 30 years	0.19	(0.39)	0.20	(0.40)
30–39 years	0.18	(0.38)	0.17	(0.38)
40–49 years	0.17	(0.38)	0.20	(0.40)
50–59 years	0.25	(0.43)	0.23	(0.42)
60 years and older	0.21	(0.41)	0.21	(0.41)
*Education*				
Lower/middle secondary schooling	0.59	(0.49)	0.62	(0.49)
Upper secondary schooling	0.38	(0.49)	0.38	(0.48)
Without school leaving certificate	0.01	(0.11)	0.00	(0.07)
In education	0.01	(0.11)	0.00	(0.05)
*Employment status*				
Full-time employment	0.49	(0.50)	0.56	(0.50)
Part-time employment	0.16	(0.36)	0.17	(0.37)
Other employment status	0.14	(0.34)	0.05	(0.23)
Not employed	0.22	(0.42)	0.21	(0.41)
*Occupation*				
White collar worker	0.46	(0.50)	0.59	(0.49)
Blue collar worker	0.13	(0.34)	0.08	(0.27)
Self-employed	0.06	(0.24)	0.04	(0.20)
Civil servant	0.05	(0.22)	0.05	(0.22)
Other occupational status	0.04	(0.20)	0.05	(0.21)
Occupation information missing	0.26	(0.44)	0.20	(0.40)
*Household characteristics and income*				
Single person HH	0.23	(0.42)	0.24	(0.43)
Number of people in HH	2.45	(1.22)	2.41	(1.19)
Children below age 3 years in HH	0.05	(0.22)	0.07	(0.25)
Children between 3 and 5 years in HH	0.05	(0.22)	0.05	(0.22)
Children between 6 and 10 years in HH	0.07	(0.25)	0.07	(0.25)
Children between 11 and 15 years in HH	0.06	(0.24)	0.07	(0.25)
No children below age 16 years in HH	0.77	(0.42)	0.74	(0.44)
Monthly net household income in euro	3172.24	(1344.12)	2826.10	(1293.52)
Household income information missing	0.05	(0.22)	0.17	(0.38)
*Satisfaction*				
General life satisfaction	7.36	(1.69)	6.95	(2.12)
Satisfaction with family life	7.80	(1.91)	6.99	(2.50)
Satisfaction with childcare	7.25	(2.23)	4.26	(2.94)
Number of observations	19430 (3036)		14781 (3054)	
Number of individuals	19430 (3036)		8977 (1925)	

*Notes:* The table shows descriptive statistics of the German Socio-Economic Panel from 2018 and the COMPASS survey from May and June 2020. Data is weighted with individual weights. Satisfaction with care is only available for individuals with children living in the household. The corresponding number of observations for satisfaction with childcare is reported in parentheses. In the COMPASS surveys, respondents were sometimes interviewed again at a later date

*Source:* Own calculations based on infratest dimap COMPASS and SOEP v35

In order to make our SOEP-subsample as comparable as possible to the COMPASS sample, we could, in principle, restrict the SOEP sample to individuals stating in previous surveys that they use the internet regularly. We focus on the online population in a robustness check (Section VI). Although this information is only available for a subset of our sample, we reach the same conclusions. To maintain a larger number of observations, we do not apply this sample restriction to our main analysis.

### Empirical strategy

Our descriptive analysis is based on graphical illustrations of the satisfaction measures between the two surveys on average and for certain sample splits. We split the sample by the age of the youngest child, principally motivated by the differential impact of day care center closures on families with children of different ages or with no children under 16 years in the household. We also present differences by parental gender and the level of secondary schooling. These splits are motivated by the literature that predicts uneven impacts of the Covid-19 crisis by gender and socioeconomic class (Alon et al. 2020; Benzeval et al. 2020; Conti 2020; Dingel and Neiman 2020; Jessen and Waights 2020; Hupkau and Perongolo 2020) as well as evidence that day care centers improve the life satisfaction of mothers but not of fathers (Diener et al. 2009; Schmitz 2020; Schober and Stahl 2016), and that enrollment rates in day care centers differ by family background in Germany (Jessen et al. 2020).^10^ Our descriptive analysis also examines the likelihood of reporting that measures are ‘strict’ and the changes in well-being by whether or not individuals report being affected by closures.

Despite the survey questions relating to satisfaction being exactly identical, there is a limitation for a direct comparison in that the different survey modes that may themselves affect the reported well-being of individuals. COMPASS was conducted online, while the regular SOEP survey is typically conducted in personal interviews. The situational context (“normal interview settings” vs. exceptional Covid-19-setting, which reminds respondents in several questions that the current situation is insecure) could also affect the general level of reported satisfaction. While a direct comparison with the SOEP data from 2018 to the COMPASS data from 2020 gives some general idea of the two data sources, it should be noted that such comparisons may include both survey mode and external context effects. Thus, our discussion of the descriptive results concentrates more on the relative changes by sub-sample rather than absolute changes. By focusing on *relative* changes, we essentially look at changes in the *distributions within each sample* and avoid context effects that could shift the levels of the outcomes.

To address the difference in survey contexts more formally, we use a difference-in-differences (DiD) design. We pool the SOEP 2018 and COMPASS 2020 data to estimate the following OLS regression model:

1 $$\begin{array}{l}Y_{it} \,=\, \alpha \,+\, \mathop {\sum}\nolimits_a {\beta _a\left( {COVID_t \,\times\, AGE_{ai}} \right)}\\ \qquad+\, \gamma COVID_t \,+\, \mathop {\sum}\nolimits_a {\delta _aAGE_{ai} \,+\, \theta X_{it} \,+\, \varepsilon _{it}} \end{array}$$

where *Y*_*it*_ is satisfaction with life in general, with family life, or with childcare for individual *i* observed at time *t*, *COVID*_*t*_ is an indicator that is equal to 1 if time period *t* belongs to the year 2020 during the Covid-19 pandemic (or, equivalently, if the observation comes from the COMPASS rather than the SOEP data), *AGE*_*ai*_ indicates the age band, *a*, of the youngest child in individual *i*’s household: either 0–2 years, 3–5 years, 6–10 years, 11–15 years with an omitted category of no children under 16 years, *X*_*it*_ is a vector of individual control variables and *ε*_*it*_ is the error term. We include controls that use the maximum possible flexibility: We include indicators for the 16 federal states, for respondent’s age in years, for their level of education (upper, middle or lower school track, no school degree, or still in education), for household size, if the respondent is female, for eleven net monthly household income categories, their employment status (full-time, part-time, not employed, others and missing information), and their occupational status (white collar, blue collar, self-employed, civil servant, others and missing information).^11^ Standard errors are clustered at the person-level, as some randomly chosen individuals are interviewed twice in the COMPASS survey.^12^

The main coefficients of interest are *β*_*a*_ which capture the differential change in satisfaction under Covid-19 for parents of dependent children in age band *a* relative to individuals without children. The parameter *γ* estimates the change under Covid-19 for individuals without dependent children, thus netting out the general well-being impacts of Covid-19 that may include impacts through fear of infections or the general impacts of lockdowns e.g., on loneliness. The parameter also nets out any trends in well-being for the general population as well as any potential context or survey impacts that are constant across individuals. The constant *α* and parameters *δ*_*a*_ captures mean satisfaction in the pre-period and *θ* are the estimates for the control variables.

**Fig. 2:**
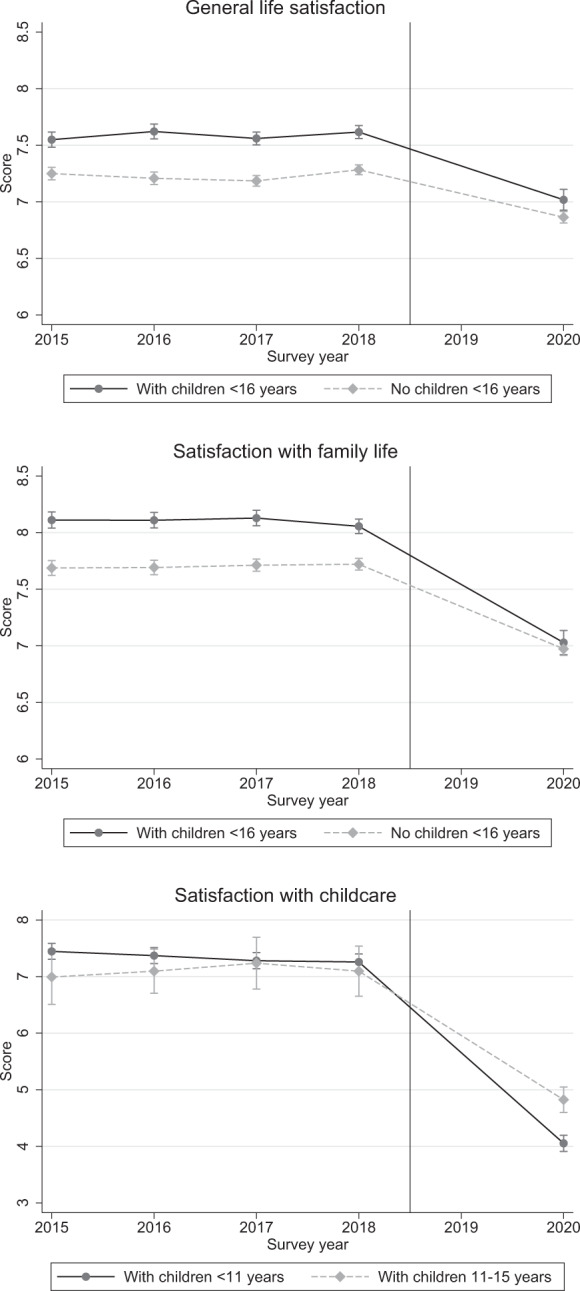
Satisfaction of individuals with and without children of specific ages in the household, 2015–2020. *Notes:* The figure shows a common trend in average satisfaction scores for 2015–2018 (based on SOEP) for individuals with and without children of specific ages in the household. Data for 2020 refer to COMPASS data collected during the Covid-19 pandemic. Data is weighted by individual weights. *Source:* Own calculations based on infratest dimap COMPASS and SOEP v35

For our DiD to be valid we must assume parallel trends, i.e., that satisfaction for individuals with dependent children and other individuals would follow the same path in the absence of Covid-19. In Fig. 2 we plot well-being for these two groups not just for 2018 and 2020 but also for the previous three waves of the SOEP (2015, 2016 and 2017). The plots show that the trends for our three satisfaction outcomes are broadly parallel in the pre-Covid-19 period. For our parallel trend assumption to hold we also require that any survey or context effects are constant across the two groups. While we are unable to test to assumption, we see no particular reason why there would be large differences in these effects for parents of dependent children.^13^

While day care and school closures are expected to be a significant mechanism for differential effects, our DiD estimate is not to be directly interpreted as an effect of the closures. Other aspects of the pandemic (e.g., fear of the virus, social distancing, and the economic shut-down) may impact on parents differently, too. In some respects parents might be expected to fare better than non-parents, for example in relation to social distancing, where people living alone or without children may experience greater loneliness. In other respects parent may fare worse, for example due to closures of certain services and facilities (e.g., playgrounds) that they are more reliant on. In the former case, our estimates might be smaller than the impact of the closures and in the latter our estimates could exceed the impacts of the closures. Table 7 reports differences between parents and non-parents in characteristics, which could help motivate further such differential effects.

We examine the closures channel in two alternative specifications that estimate heterogeneous DiD effects: one that shows the effects for May and June, separately, where in the latter month schools and day care centers had begun to reopen, and another that shows effect for parents reporting either being affected or not affected by the closures. Parents reporting not being affected by closures are most likely those receiving emergency care due to working in a systemically relevant occupation. A weakness of these specifications is that change in child care availability coincides with other changes (e.g., working in a systemically relevant occupation may be more stressful), however, they hope to provide some insight on the closures channel.

## Results

### Changes in satisfaction under Covid-19

**Fig. 3:**
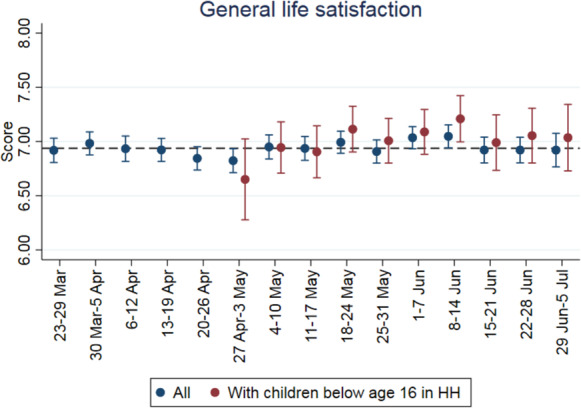
General life satisfaction for individuals with and without children during the Covid-19 pandemic. *Notes:* The dashed horizontal line represents the mean value of all individuals in the observation period. *Source:* Own calculations based on infratest dimap COMPASS

**Fig. 4:**
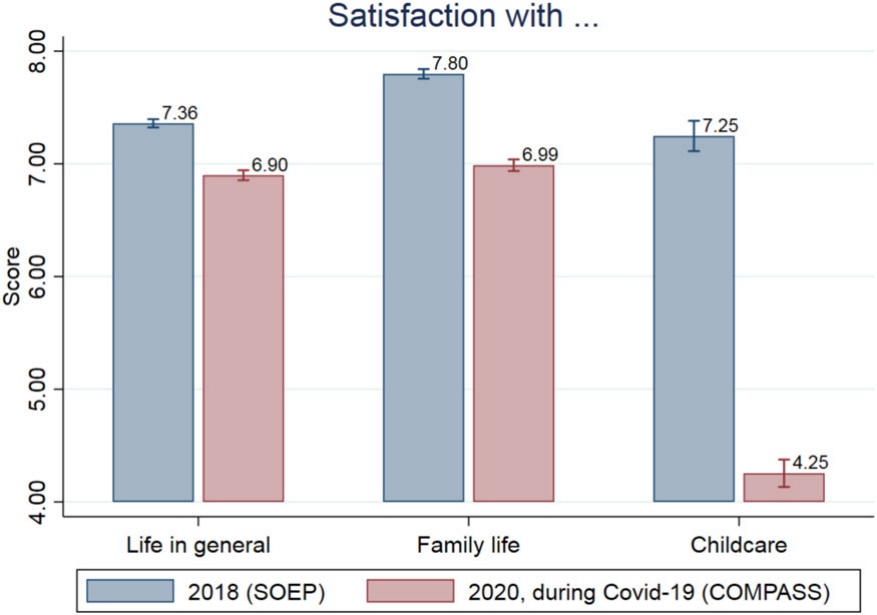
Satisfaction with life in general, family life and childcare in 2018 and 2020 during the Covid-19 pandemic. *Source:* Own calculations based on infratest dimap COMPASS and SOEP v35

**Fig. 5:**
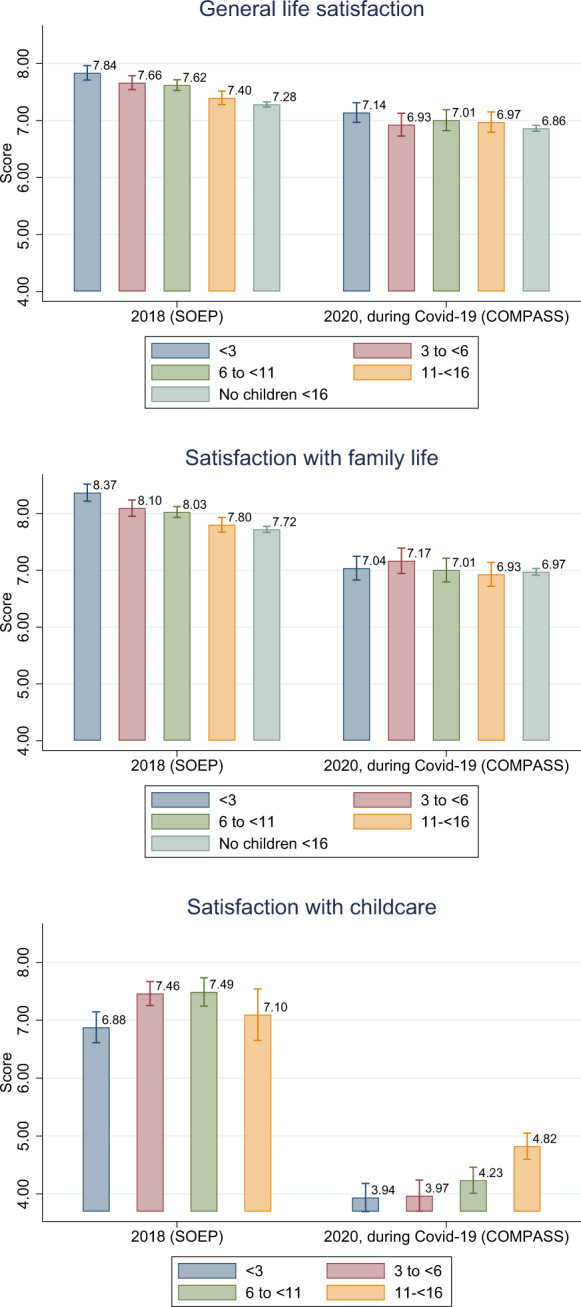
Satisfaction by age of the youngest child in the household before and during the Covid-19 pandemic. *Source:* Own calculations based on infratest dimap COMPASS and SOEP v35

Figure 3 plots the week-by-week evolution of general life satisfaction during Covid-19 for all individuals and for individuals with dependent children, the latter of which starts midway when information on children began to be collected.^14^ Life satisfaction appears to move in response to Covid-19 restrictions: it is at its lowest at the end of April when infections had been low for some time but the lockdown was still in effect. Satisfaction begins to recover somewhat in May and June as restrictions are eased and this recovery is relatively stronger for individuals with children. In Fig. 4, we plot the sample means for all individuals interviewed in the Covid-19 period and in 2018 from the SOEP survey. Both general life satisfaction and satisfaction with family life are lower in the Covid-19 survey, by 0.5 and 0.8 points, respectively. Satisfaction with childcare (asked only of individuals with dependent children) is 3 points lower under Covid-19, representing an even larger difference.^15^ In Fig. 5, we make the same comparison between surveys, this time splitting the sample by the age of the youngest child in the household. In pre-Covid-19 times, life satisfaction and satisfaction with family life is highest among respondents with very young children, decreasing as the age of the child increases. However, during Covid-19, life satisfaction and satisfaction with family life are at comparable levels irrespective of the age of the youngest child. Correspondingly, the largest decreases under Covid-19 are seen for families with young children (toddlers and preschoolers). In terms of satisfaction with childcare, under Covid-19 the level is lowest for respondents with young children and increases with child age. Again, compared with the 2018 sample, the distribution of satisfaction has changed in a way that marks a relative decline for those with younger children. This is presumably because older children are more rarely cared for institutionally and can work independently on schoolwork.^16^

**Fig. 6:**
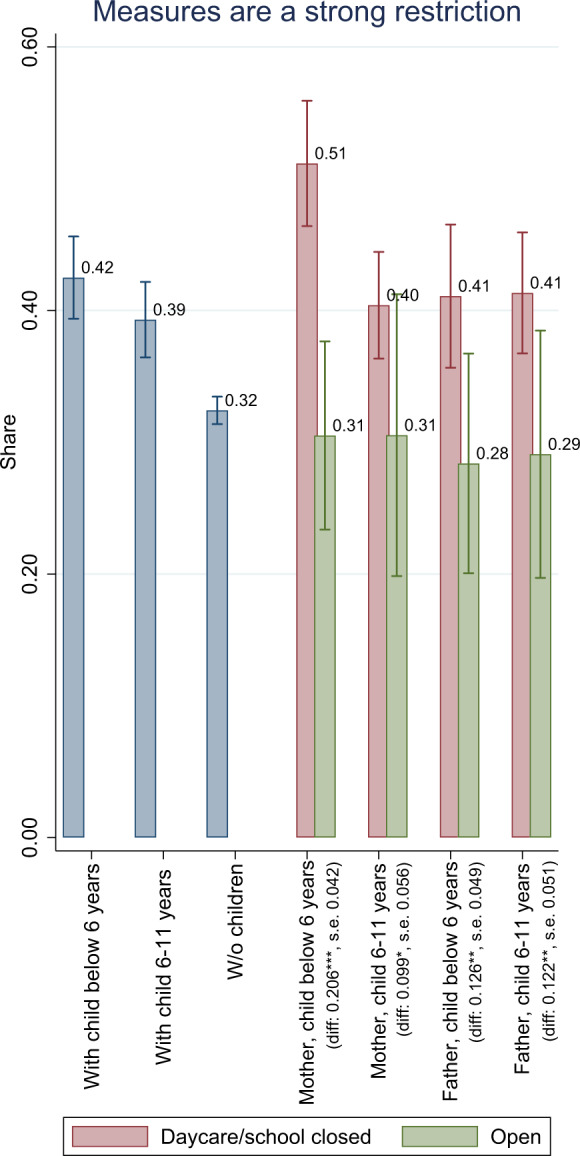
Measures taken to contain Covid-19 perceived as a strong restriction. *Notes:* Are public measures taken to contain Covid-19 perceived as a strong restriction? We first show general agreement to this question by age of the youngest child in the household, and then differentiate by gender and whether the person reports to be affected by daycare and school closures. ***p < 0.01; **p < 0.05; *p < 0.1 refers to the statistical significance of group differences. *Source:* Own calculations based on infratest dimap COMPASS

Overall, the reported levels of satisfaction with life in general, with family life, and with childcare are significantly lower during Covid-19. However, it is also apparent that the changes are dependent on as the presence of young children. A likely explanation for this heterogeneity is the closure of schools and day care centers. In Fig. 6, we show whether respondents perceive the measures taken to contain Covid-19 as very severe restrictions. About 42 percent of people with day care-aged children and 39 percent with school-aged children perceive the measures as very severe. Among respondents without children, this share is only 32 percent. We further differentiate by the actual exposure to day care and school closures. Parents who are unaffected by the closures appear similar to individuals without children in their likelihood to report the measures are strict (around 30 percent) whereas parents who are affected are much more likely to report measures as being strict, especially mothers of children under 6 years: 51 percent do so. This suggests that day care and school closures could be a major component of the differential impact of the restrictions on parents.

### Difference-in-differences estimates

Table 2 reports the results of the DiD analysis outlined in Eq. 1. Columns (1) and (2) report the results for life satisfaction with and without controls, columns (3) and (4) do the same for satisfaction with family life, and columns (5) and (6) do so for satisfaction with childcare. Including controls substantially increases the explanatory power of the model (the R² increases roughly from 0.01 to about 0.1) but the coefficient estimates remain fairly stable. The Covid-19 variable shows significant decreases in satisfaction for the first two outcomes since 2018 for the control group, i.e., those without dependent children, and for those with children aged 11–15 years for satisfaction childcare. As discussed, this includes the general impact of Covid-19 and restrictions, but it could also include any unrelated trends between 2018 and 2020 as well as any survey and context effects resulting from the change of dataset¸ thus it cannot necessarily be interpreted as a Covid-19 effect. Nevertheless, such a large drop in satisfaction with childcare for the 11–15 group compared with the other satisfaction measures is difficult to explain without considering school closures affecting these children. The interactions with the age of the youngest child show significant negative changes in all three measures of satisfaction for parents of younger children (under 11 years) compared with the control groups. As discussed, these changes should capture the differential impact of restrictions on families with younger children, in the most part due to day care and school closures.

**Table 2:** Changes in parental well-being (difference-in-differences)

	Satisfaction with ...					
	Life in general		Family life		Childcare	
	(1)	(2)	(3)	(4)	(5)	(6)
Covid-19	−0.41*** (0.03)	−0.33*** (0.04)	−0.73*** (0.04)	−0.67*** (0.05)	−2.31*** (0.27)	−2.56*** (0.27)
Covid-19 × child below 3 years	−0.24** (0.12)	−0.41*** (0.12)	−0.57*** (0.14)	−0.64*** (0.15)	−0.65** (0.33)	−0.42 (0.33)
Covid-19 × child 3–5 years	−0.33*** (0.13)	−0.34*** (0.12)	−0.16 (0.14)	−0.24* (0.14)	−1.20*** (0.32)	−0.93*** (0.32)
Covid-19 × child 6–10 years	−0.19* (0.11)	−0.19* (0.11)	−0.28** (0.12)	−0.32** (0.13)	−1.07*** (0.31)	−0.90*** (0.30)
Covid-19 × child 11–15 years	−0.03 (0.12)	0.03 (0.12)	−0.12 (0.13)	−0.12 (0.14)		
Child below 3 years	0.52*** (0.08)	0.63*** (0.10)	0.63*** (0.09)	0.35*** (0.11)	−0.24 (0.28)	−0.45 (0.29)
Child 3–5 years	0.39*** (0.07)	0.33*** (0.09)	0.36*** (0.08)	0.09 (0.10)	0.34 (0.26)	0.07 (0.26)
Child 6–10 years	0.34*** (0.06)	0.28*** (0.08)	0.31*** (0.06)	0.04 (0.08)	0.48* (0.26)	0.35 (0.25)
Child 11–15 years	0.14** (0.07)	0.07 (0.08)	0.07 (0.08)	−0.18** (0.09)		
No. of observations	34,296	34,296	31,990	31,990	5764	5764
*R*^2^	0.008	0.102	0.008	0.095	0.021	0.103
Control variables		✓		✓		✓

*Notes:* The table reports regression results of the difference-in-differences model outlined in eq. (1). Robust standard errors allow for clustering at the individual level and are reported in parentheses. If indicated, control variables are included (dummies for all categories of the following variables: federal state, household size, age in years, education, gender, household income, occupation, employment status

*Source*: Own calculations based on infratest dimap COMPASS and SOEP v35

***p < 0.01; **p < 0.05; *p < 0.1

Interestingly, those individuals with children aged 11–15 years do not experience significantly different changes in satisfaction with life in general or with family life compared to individuals without children under 16 years of age. This may suggest that school closures for older children are less detrimental to the well-being of their parents or, at least, that the costs of homeschooling are almost netted out by the benefits of spending more time with children for the average parent. For younger ages, we see the largest dissatisfaction with childcare and with life in general for the parents of children aged 3–5, which is consistent with the high pre-crisis usage of day care centers and the high level of parental input required in looking after children in this age group.

For younger children (0–2), the decrease in satisfaction with childcare is larger than that for the unaffected group, but not to a statistically significant degree in the model with controls. Perhaps this reflects that childcare centers are attended by about 34 percent of children younger than three, and 96 percent of children between three and six (Autorengruppe Bildungsberichterstattung 2020). Nevertheless, the under threes group sees the largest drops in satisfaction with family life and large drops in satisfaction with life in general, suggesting that, where parents of children in this age group are affected by closures of day care centers, the well-being impacts are significant. Finally, the effects for parents of children aged 6–10 are similar to the effects for children aged 3–5, albeit with smaller decrease in life satisfaction, perhaps due to the 6–10 age group requiring somewhat less parental input than the 3–5 age group.

In Table 3, we examine effect heterogeneity. For simplicity, we now measure the average effect across all age groups by using one variable for people with children between 0 and 15 years of age (or between 0 and 10 for the childcare variable). The decreases in satisfaction are larger for parents surveyed in the earlier part of the survey window (covering most of the month of May, see column 2) compared with the later part (June, see column 3), in line with the gradual reopening of schools and day care centers. If we differentiate directly by whether parents were affected by day care and school closures, we also find larger reductions in satisfaction with life and childcare for affected parents. For satisfaction with family life, the differences are not so clear, suggesting that other factors contribute to the reduction in satisfaction with family life that apply equally to both groups (e.g., a lack of ‘playdates’, meeting grandparents, and closures of playgrounds) or, alternatively, that emergency workers that have priority access to day care are burdened by their workload and/or associated stress in a way that impact negatively or their family lives. Either way, these specification confirm that the closures are a major channel at least for two of the satisfaction outcomes.

**Table 3:** Heterogeneities in parental well-being changes

		Heterogeneity by ...							
		Interview date		Day care/school closure		Education		Gender	
	All	May	June	Affected	Not affected	High	Low	Female	Male
	(1)	(2)	(3)	(4)	(5)	(6)	(7)	(8)	(9)
*Satisfaction with life in general*									
Covid-19	−0.33*** (0.04)	−0.32*** (0.04)	−0.30*** (0.04)	−0.33*** (0.04)	−0.33*** (0.04)	−0.46*** (0.06)	−0.25*** (0.05)	−0.36*** (0.06)	−0.32*** (0.05)
Covid-19 × child below 16 years	−0.20*** (0.07)	−0.28*** (0.07)	−0.17** (0.07)	−0.24*** (0.07)	−0.04 (0.12)	−0.16* (0.10)	−0.23** (0.09)	−0.25*** (0.10)	−0.10 (0.09)
Child below 16 years	0.28*** (0.06)	0.28*** (0.06)	0.28*** (0.06)	0.28*** (0.06)	0.28*** (0.06)	0.31*** (0.10)	0.27*** (0.09)	0.30*** (0.09)	0.28*** (0.09)
Pre-Covid-19 (2018)	6.88*** (0.43)	6.88*** (0.44)	6.88*** (0.44)	6.88*** (0.43)	6.88*** (0.43)	7.81*** (0.45)	6.40*** (0.58)	7.79*** (0.81)	6.45*** (0.55)
No. of observations	34,296	24,683	24,721	33,710	31,810	12,288	21,348	18,191	16,105
*R*^2^	0.101	0.101	0.101	0.101	0.101	0.090	0.115	0.099	0.131
*Satisfaction with family life*									
Covid-19	−0.68*** (0.05)	−0.73*** (0.05)	−0.62*** (0.05)	−0.68*** (0.05)	−0.68*** (0.05)	−0.80*** (0.07)	−0.59*** (0.06)	−0.61*** (0.07)	−0.74*** (0.06)
Covid-19 × child below 16 years	−0.32*** (0.08)	−0.35*** (0.09)	−0.28*** (0.09)	−0.32*** (0.08)	−0.30** (0.13)	−0.17 (0.12)	−0.40*** (0.10)	−0.38*** (0.11)	−0.24** (0.11)
Child below 16 years	0.02 (0.07)	0.02 (0.07)	0.02 (0.07)	0.02 (0.07)	0.02 (0.07)	0.05 (0.12)	0.03 (0.09)	−0.09 (0.10)	0.18* (0.10)
Pre-Covid-19 (2018)	6.21*** (0.44)	6.21*** (0.45)	6.21*** (0.45)	6.21*** (0.44)	6.21*** (0.44)	6.70*** (0.78)	5.66*** (0.57)	6.83*** (0.76)	5.90*** (0.58)
No. of observations	31,990	24,507	24,545	31,404	29,504	11,536	19,826	17,017	14,973
*R*^2^	0.094	0.094	0.094	0.094	0.094	0.104	0.111	0.085	0.133
*Satisfaction with childcare*									
Covid-19	−2.27*** (0.25)	−2.39*** (0.27)	−2.15*** (0.27)	−2.34*** (0.26)	−1.89*** (0.37)	−2.75*** (0.40)	−2.14*** (0.33)	−2.19*** (0.30)	−2.39*** (0.40)
Covid-19 × child below 11 years	−0.93*** (0.27)	−1.18*** (0.29)	−0.69** (0.29)	−1.08*** (0.28)	−0.46 (0.41)	−0.48 (0.42)	−1.10*** (0.36)	−1.07*** (0.33)	−0.75* (0.43)
Child below 11 years	0.16 (0.24)	0.16 (0.24)	0.16 (0.24)	0.16 (0.24)	0.16 (0.24)	−0.17 (0.35)	0.25 (0.32)	0.08 (0.28)	0.24 (0.38)
Pre-Covid-19 (2018)	7.10*** (0.23)	7.10*** (0.23)	7.10*** (0.23)	7.10*** (0.23)	7.10*** (0.23)	7.62*** (0.34)	6.91*** (0.30)	7.16*** (0.26)	7.04*** (0.36)
No. of observations	6108	4578	4566	5522	3622	2461	3218	3345	2763
*R*^2^	0.089	0.088	0.088	0.089	0.088	0.160	0.114	0.135	0.164
Control variables	✓	✓	✓	✓	✓	✓	✓	✓	✓

*Notes:* The table reports regression results of the difference-in-differences model outlined in eq. (1) estimated separately for the subgroups. “High education” refers to individuals with upper secondary school leaving certificates, “low education” refers to individuals with low and middle secondary school leaving certificates. The coefficient on “Pre-Covid-19 (2018)” refers to the conditional mean of individuals without dependent children in the 2018 SOEP data. Included control variables are described in the notes to Table 2. Robust standard errors allow for clustering at the individual level and are reported in parentheses

Source: Own calculations based on infratest dimap COMPASS and SOEP v35

***p < 0.01; **p < 0.05; *p < 0.1

Parents with higher educational attainment generally see smaller relative changes compared to parents with lower educational attainment, especially in satisfaction with childcare and with family life. Parents with lower educational attainment may find the extra childcare and homeschooling more difficult if, for example, they are less able to work from home in their jobs, if they are generally more time-constrained, or if they lack resources to provide educational activities at home. Finally, mothers see larger decreases in satisfaction with family life and with life in general than do fathers, although for changes in satisfaction with childcare, this pattern is reversed. This could be explained by the observation that fathers affected by closures show a larger decrease in satisfaction with childcare than mothers affected by closures if they have school aged children (for details, see Huebener et al. 2020).

### Robustness checks

**Fig. 7:**
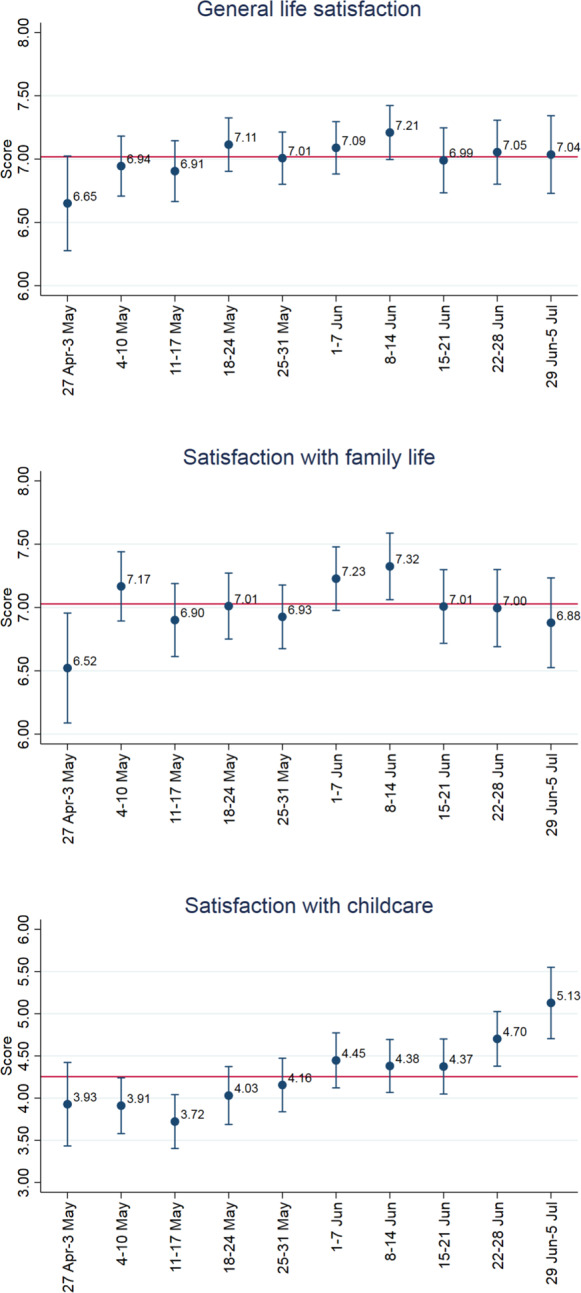
Parental satisfaction over the course of the Covid-19 pandemic. *Notes:* The figure shows the evolution of satisfaction with life in general, family life and childcare in May and June 2020 for parents with children below age 16 living in the household. The red horizontal line represents the mean value of all individuals in the observation period. *Source:* Own calculations based on infratest dimap COMPASS

In this section, we evaluate the robustness of our findings. First, we address concerns related to the online survey of well-being measures during Covid-19 in the COMPASS data. In our main analysis, this data is compared to all available SOEP data from 2018. As the regular SOEP is mainly conducted in face-to-face interviews, the COMPASS online survey may systematically distort the results as it captures a slightly different population. Based on previous SOEP surveys from 2003, 2008, and 2013, we have information on use of the internet (see Appendix Table 5) for 64 percent of individuals in our SOEP data for 2018. Of those respondents, 59 percent of our sample report that they use the internet daily and another 23 percent at least once a week. In Fig. 7, we examine whether the use of the internet correlates systematically with satisfaction with life in general, family life, and childcare. While satisfaction is very similar across individuals who use the internet rarely or regularly, satisfaction tends to be lower for individuals who never use the internet. However, on average, it is higher for individuals without information on the use of internet. To check the sensitivity of our findings to the focus on potentially different populations (COMPASS, excluding the offline population), we restrict the 2018-SOEP data in the DiD analysis to individuals who use the internet at least once a week. Compared to our main findings (Table 4, column 1), the results are very similar if we drop individuals with no information on their use of the internet and individuals that use the internet less than once a week (Table 4, column 2).

**Table 4:** Robustness checks

	Main	Only online population	z-standardized outcomes	log outcome	Comparison to 2017
	(1)	(2)	(3)	(4)	(5)
*Satisfaction with life in general*					
Covid-19	−0.33*** (0.04)	−0.22*** (0.05)	0.06*** (0.02)	−0.06*** (0.01)	−0.25*** (0.04)
Covid-19 × child below 3 years	−0.41*** (0.12)	−0.41** (0.16)	−0.28*** (0.07)	−0.07*** (0.02)	−0.61*** (0.12)
Covid-19 × child 3–5 years	−0.34*** (0.12)	−0.40*** (0.14)	−0.21*** (0.06)	−0.05** (0.02)	−0.23* (0.13)
Covid-19 × child 6–10 years	−0.19* (0.11)	−0.15 (0.12)	−0.13** (0.06)	−0.03 (0.02)	−0.17 (0.11)
Covid-19 × child 11–15 years	0.03 (0.12)	0.05 (0.13)	0.00 (0.06)	−0.01 (0.02)	−0.02 (0.12)
No. of observations	34,296	26,185	34,296	34,085	32,931
*R*^2^	0.102	0.116	0.102	0.096	0.113
*Satisfaction with family life*					
Covid-19	−0.67*** (0.05)	−0.59*** (0.06)	0.07*** (0.02)	−0.12*** (0.01)	−0.68*** (0.05)
Covid-19 × child below 3 years	−0.64*** (0.15)	−0.56*** (0.18)	−0.34*** (0.07)	−0.10*** (0.02)	−0.83*** (0.15)
Covid-19 × child 3–5 years	−0.24* (0.14)	−0.26 (0.16)	−0.15** (0.06)	−0.03 (0.02)	−0.29** (0.14)
Covid-19 × child 6–10 years	−0.32** (0.13)	−0.37*** (0.13)	−0.17*** (0.05)	−0.05** (0.02)	−0.33*** (0.13)
Covid-19 × child 11–15 years	−0.12 (0.14)	−0.05 (0.15)	−0.06 (0.06)	−0.03 (0.02)	−0.23 (0.14)
No. of observations	31,990	23,984	31,990	31,643	30,629
*R*^2^	0.095	0.097	0.095	0.086	0.104
*Satisfaction with childcare*					
Covid-19	−2.56*** (0.27)	−2.42*** (0.30)	0.13 (0.12)	−0.46*** (0.05)	−1.84*** (0.24)
Covid-19 × child below 3 years	−0.42 (0.33)	−0.63* (0.37)	−0.09 (0.14)	−0.06 (0.06)	−1.35*** (0.30)
Covid-19 × child 3–5 years	−0.93*** (0.32)	−1.10*** (0.35)	−0.32** (0.13)	−0.13** (0.06)	−1.62*** (0.29)
Covid-19 × child 6–10 years	−0.90*** (0.30)	−1.16*** (0.33)	−0.34*** (0.13)	−0.17*** (0.06)	−1.70*** (0.28)
No. of observations	5764	4725	5764	5328	5875
*R*^2^	0.103	0.159	0.101	0.099	0.169
Control variables	✓	✓	✓	✓	✓

*Notes:* The table reports regression results of the difference-in-differences model outlined in eq. (1). The z-standardized outcomes (for column 3) are standardized by survey year. Robust standard errors allow for clustering at the individual level and are reported in parentheses. Control variables as described in the notes to Table 2

Source: Own calculations based on infratest dimap COMPASS and SOEP v35

***p < 0.01; **p < 0.05; *p < 0.1

In our DiD approach, we use individuals with no dependent children (or comparably older children) as a control group to account for level shifts due to trends or potential mode and context effects. Alternatively, we could also standardize the outcomes by sample to mean zero and standard deviation of one (i.e., *z*-transformation), such that at each point in time, satisfaction of individuals is compared to the sample mean in the respective period. Any general differences in means, as well as in the dispersion of satisfaction, are removed. While the resulting estimates remove potential common mode and context effects, the estimates could underestimate the true impact of Covid-19 on satisfaction, because strongly affected, larger groups would pull down the sample mean that we remove. On the other hand, standardizing outcomes improves the comparability of effect sizes across studies. Our results show that standardizing the outcomes by survey wave generates very similar patterns across children’s age but, as expected, removes most of the Covid-19 level shift (Table 4, column 3). The relative changes in life satisfaction when expressed in standard deviations are −0.21 and −0.28 for parents with a children aged below 3 and 3–5 years, respectively. Given we know that our effects for mothers only are a little higher, these effect sizes are comparable in magnitude to the increase in life satisfaction of 0.30 standard deviations found by Schmitz (2020) for mothers that receive a day care place in Germany.

Our analysis shows that having children is associated with a higher life satisfaction in levels. While this is consistent with a literature that shows having children increases life satisfaction (e.g., Myrskylä and Margolis 2014), it is important to demonstrate that the greater changes we observe for parents during Covid-19 are not simply proportional decreases based on a higher starting point (Kahn-Lang and Lang 2020). In column (4), therefore we estimate a specification that uses life satisfaction in logs as the outcome. The significant decreases here are consistent with prior results showing our results are not driven by this aspect of functional form.^17^

In our main analysis, we compare satisfaction during Covid-19 (COMPASS) to the earliest available pre-Covid-19 data (SOEP 2018). To rule out that our results depend on the choice of the reference year to represent “normal times”, we also consider SOEP 2017 as the reference point. As satisfaction levels do not vary much in normal times, the results are, as expected, very similar (Table 4, column 5).

## Discussion and conclusion

This study examines the possible differential impacts of the Covid-19 outbreak, and its related restrictions, on the well-being of individuals with dependent children in Germany using a new dataset of well-being for Germany, the COMPASS survey. We look at May and June, 2020, when new infection rates in Germany were low and the majority of restrictions were relaxed, but when schools and day care centers were still closed to most children. Using a combination of descriptive analyses and a difference-in-differences design, we find satisfaction with life overall, with family life, and with childcare decreased under Covid-19 by more for individuals with children than for other individuals. We find the relative decrease to be greatest for respondents with children under 11 years of age, for women, and for respondents with a lower secondary schooling degree. Our results are robust to several checks. The closures of schools and day care centers is a prominent explanation for these relative decreases of parental well-being.

We find extra decreases in satisfaction for parents that are similar in size or larger to estimates of the overall effects of Covid-19 on well-being in other countries (e.g., Adams-Prassl et al. 2020b; Etheridge and Spantig 2020) and similar in size to the positive effects of getting a day care spot on maternal well-being (Schmitz 2020). The effects are about half the size of the negative impact on well-being of a job loss (Kassenboehmer and Haisken-DeNew 2009). Such significant drops in well-being may have detrimental impacts on other important outcomes, such as child development, family stability, and the labor force productivity of parents (e.g., Frank and Gertler 1991; Smith 2004; Oswald et al. 2015). While the drop in well-being we record may be partly temporary (if mostly linked to contemporaneous restrictions), some parts of the direct effect and many of the indirect effects may be permanent.

Our estimates represent an important consideration when determining optimal lockdown policy during the ongoing or possible future pandemics. For example, in combination with other information (e.g., on the way viruses spread in schools and on other economics outcomes), policymakers may decide to prioritize keeping schools and day care centers open over other public settings/places like bars and restaurants. Furthermore, from our estimates, important conclusions can be drawn regarding the potential need for extra support for parents. For example, financial benefits during a pandemic may alleviate stress by covering earning losses arising from reducing hours. Moreover, the provision of family counseling may help avoid some negative outcomes for families and children. In this respect, it would be advisable for crisis teams at regional and national levels, from the beginning of a pandemic, to include not only virologists, medical experts in general, and economists, but also representatives of family and education policy experts.

## Figures and Tables

**Fig. 8:**
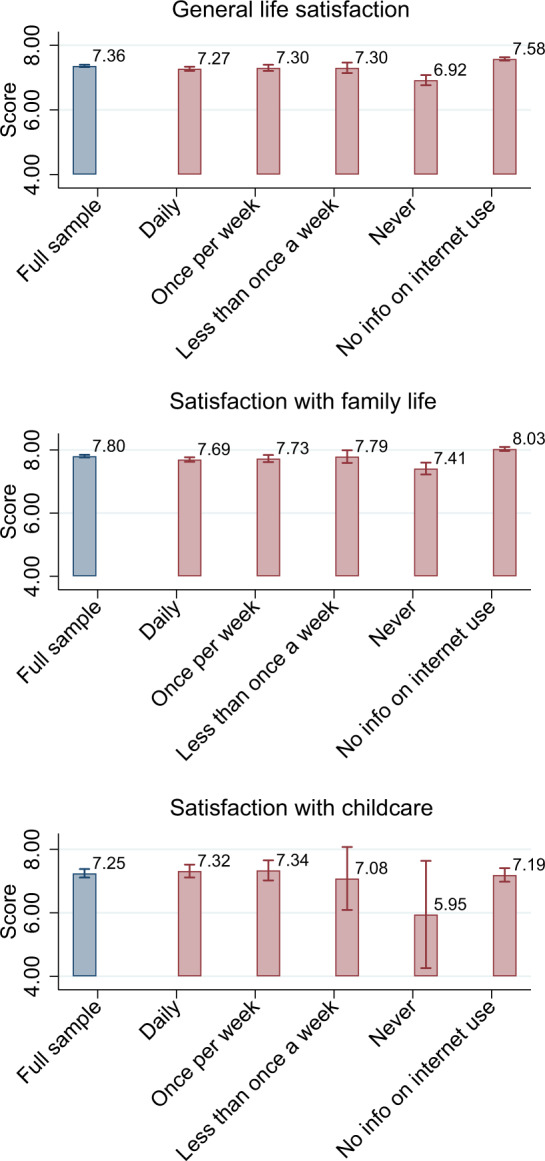
Satisfaction by internet usage in the SOEP. *Source:* Own calculations based on SOEP v35

## Appendix

Tables 5–7

**Table 5:** Descriptive statistics, unweighted

	2018 (SOEP v35)		2020 (COMPASS)	
	Mean	(SD)	Mean	(SD)
*Individual characteristics*				
Female	0.54	(0.50)	0.52	(0.50)
Age in years	45.14	(14.71)	49.19	(14.09)
Below 30 years	0.19	(0.39)	0.13	(0.33)
30–39 years	0.16	(0.37)	0.15	(0.36)
40–49 years	0.21	(0.41)	0.17	(0.37)
50–59 years	0.24	(0.43)	0.25	(0.43)
60 years and older	0.19	(0.40)	0.30	(0.46)
*Education*				
Lower/middle secondary schooling	0.58	(0.49)	0.66	(0.47)
Upper secondary schooling	0.39	(0.49)	0.33	(0.47)
Without school leaving certificate	0.01	(0.12)	0.00	(0.07)
In education	0.02	(0.13)	0.00	(0.04)
*Employment status*				
Full-time employment	0.44	(0.50)	0.52	(0.50)
Part-time employment	0.18	(0.39)	0.16	(0.36)
Other employment status	0.15	(0.35)	0.04	(0.20)
Not employed	0.23	(0.42)	0.27	(0.45)
*Occupation*				
White collar worker	0.45	(0.50)	0.54	(0.50)
Blue collar worker	0.12	(0.33)	0.08	(0.26)
Self-employed	0.06	(0.24)	0.04	(0.20)
Civil servant	0.05	(0.22)	0.04	(0.21)
Other occupational status	0.04	(0.20)	0.04	(0.19)
Occupation information missing	0.27	(0.44)	0.26	(0.44)
*Household characteristics and income*				
Single person HH	0.14	(0.35)	0.24	(0.43)
Number of people in HH	2.88	(1.42)	2.25	(1.08)
Children below age 3 years in HH	0.06	(0.23)	0.05	(0.23)
Children between 3 and 5 years in HH	0.06	(0.25)	0.04	(0.20)
Children between 6 and 10 years in HH	0.13	(0.34)	0.06	(0.23)
Children between 11 and 15 years in HH	0.11	(0.32)	0.06	(0.23)
No children below age 16 years in HH	0.64	(0.48)	0.79	(0.40)
Monthly net household income in euro	3285.71	(1318.26)	2796.45	(1285.25)
Household income information missing	0.04	(0.20)	0.18	(0.38)
*Satisfaction*				
General life satisfaction	7.44	(1.67)	7.00	(2.13)
Satisfaction with family life	7.90	(1.86)	7.06	(2.51)
Satisfaction with childcare	7.33	(2.18)	4.29	(2.93)
Number of observations	19430 (3036)		14781 (3054)	
Number of individuals	19430 (3036)		8977 (1925)	

*Notes:* The table shows *unweighted* descriptive statistics of the German Socio-Economic Panel from 2018 and the COMPASS survey from May and June 2020. The corresponding number of observations for satisfaction with childcare is reported in parentheses. In the COMPASS surveys, respondents were sometimes interviewed again at a later date

*Source:* Own calculations based on infratest dimap COMPASS and SOEP v35

**Table 6:** Internet use of individuals in the SOEP data Mean (s.d.)

	Mean	(s.d.)
No information on internet use	0.36	(0.48)
*With information on internet use*		
Daily use	0.59	(0.49)
At least once a week	0.23	(0.42)
At least once a month or less	0.07	(0.25)
Never	0.12	(0.32)

*Notes:* The table shows descriptive statistics of the use of the internet of respondents in German Socio-Economic Panel with information on life satisfaction in 2018. Data is weighted with individual weighs. Information on internet use results from specific survey questions in 2003, 2008 and 2013 (latest available information considered)

*Source:* Own calculations based on SOEP v35

**Table 7:** Descriptive statistics for individuals with and without dependent children

	Household with children below 16 years			
	Yes		No	
	Mean	(s.d.)	Mean	(s.d.)
	(1)	(2)	(3)	(4)
*Satisfaction*				
Satisfaction with life in general	7.62	(1.53)	7.28	(1.73)
Satisfaction with family life	8.06	(1.69)	7.72	(1.97)
Satisfaction with childcare	7.25	(2.23)	–	
*Demographics and income*				
Female	0.52	(0.50)	0.48	(0.50)
Age in years	39.25	(8.93)	47.46	(15.71)
Age below 25 years	0.07	(0.26)	0.11	(0.32)
Age between 25 and below 35 years	0.18	(0.39)	0.17	(0.38)
Age between 35 and below 45 years	0.45	(0.50)	0.09	(0.28)
Age between 45 and below 55 years	0.26	(0.44)	0.20	(0.40)
Age between 55 and below 65 years	0.03	(0.16)	0.29	(0.45)
Age 65 and older	0.00	(0.05)	0.14	(0.35)
Net household income in euro	3682.00	(1129.96)	3019.88	(1365.37)
*Education*				
Lower/middle secondary schooling	0.53	(0.50)	0.61	(0.49)
Upper secondary schooling	0.44	(0.50)	0.37	(0.48)
No schooling degree	0.02	(0.13)	0.01	(0.10)
In Education	0.01	(0.12)	0.01	(0.11)
Education information missing	0.10	(0.29)	0.10	(0.30)
*Occupation*				
White collar worker	0.54	(0.50)	0.43	(0.50)
Blue collar worker	0.13	(0.33)	0.13	(0.34)
Self-employed	0.06	(0.23)	0.06	(0.24)
Civil servant	0.06	(0.24)	0.04	(0.21)
Other occupational status	0.02	(0.14)	0.05	(0.21)
Occupation information missing	0.19	(0.39)	0.28	(0.45)
*Employment status*				
Working	0.78	(0.41)	0.69	(0.46)
Not working	0.08	(0.27)	0.22	(0.41)
On parental leave	0.09	(0.28)	0.00	(0.04)
In professional training	0.05	(0.22)	0.08	(0.27)
Pensioner	0.00	(0.03)	0.02	(0.13)

*Notes:* The table reports weighted sample means for individuals in 2018

*Source:* Own calculations based on SOEP v35

Figures 7, 8

## Footnotes

1 For the relevant age-group, online access is near universal meaning that coverage problems are not an issue. We further discuss the population with online access in the data section. We also show that our results are robust to excluding individuals without online access from the (pre-Covid-19) SOEP sample. For further details on data collection during Covid-19 in Germany, see, e.g., https://www.ratswd.de/en/topics/corona-pandemic. For another study using the COMPASS data, see Wagner et al. (2020).

2 Since April 2020, a special survey by the German Socio-Economic Panel has also raised general life satisfaction and satisfaction with family life. First results of this survey are published in Liebig (2020) and Entringer and Kroeger (2020). Moreover, the Federal Institute for Population Research (*Bundesinstitut für Bevölkerungsforschung*) analysed the well-being of parents between 17 and 24 April 2020, i.e., mainly during the lockdown. The study is based on an online survey. See Bundesinstitut für Bevölkerungsforschung (2020) for details.

3 See, e.g., a commentary by 43 female scientists of April 15, 2020: Commentary on the ad hoc statement of the National Academy of Sciences Leopoldina “Coronavirus-Pandemie—Die Krise nachhaltig überwinden” vom 13.04.2020, https://sync.academiccloud.de/index.php/s/MBO8UMvnCSwNOZe.

4 These numbers are based on responses by the interviewees that they are affected by school or day care center closures. Not affected were, e.g., families with a right to emergency care or families in which children do not yet attend a day care center.

5 In contrast, Busse and Gathmann (2018) find no effects on maternal employment either at the intensive or extensive margins for fee reductions in Germany. See Baker et al. (2008) for international evidence.

6 For details, see https://www.infratest-dimap.de/umfragen-analysen/bundesweit/coronacompass/coronacompass/.

7 This is the earliest date on which questions on satisfaction with family life and childcare were added to the COMPASS survey.

8 Individuals living with a dependent child are not necessarily parents and could instead be guardians or simply adults co-habiting with children. Nevertheless, the majority of these individuals are the child’s parents and the remainder are likely to play some role in the role in the child’s upbringing. Similarly, individuals not living with a dependent child are not necessarily non-parents as they may have older children in the household or children who have moved out. However, they are likely to be relatively less affected in terms of childcare responsibility resulting from the closures of schools and day care centers.

9 If survey participation positively correlates with well-being, such that more satisfied individuals were more likely to participate in the COMPASS survey during Covid-19, we might even underestimate well-being effects.

10 Another motivation to examine such heterogeneity is evidence on pre-existing gaps in well-being, e.g., by socioeconomic class (Powdthavee et al. 2015).

11 In the COMPASS data, information on household income, employment status and occupation are based on base data of members in the Payback Panel from which survey participants in COMPASS were recruited, i.e., the information was collected prior to the Covid-19 pandemic.

12 For the SOEP data, we consider the survey from 2018 in which each respondent was interviewed once.

13 Further, our pattern of results later appears much more consistent with differential impacts due to Covid-19 and related restrictions and would be very difficult to interpret as differential survey/context effects.

14 Figure 7 in the appendix plots the evolution of the three different satisfaction measures for individuals with children younger than 16 in the household.

15 Preliminary results for the SOEP in 2019 show similar values to those in 2018 (see Liebig 2020): The mean general life satisfaction in 2019 was 7.5, and satisfaction with family life 7.8. The values for 2019 are not statistically significantly different from the values in 2018. However, since the data from the SOEP 2019 survey are not yet available to the scientific community, our comparisons refer to the data for 2018. In addition to the COMPASS data, the SOEP-CoV survey also covers well-being during the Covid-19 crisis. For the month of April, the average life satisfaction level was 7.4, which is only slightly below the 2018 value (Liebig 2020, Entringer and Kröger 2020). The average satisfaction with family life is 7.5 points, which is 0.3 points lower than in 2018. The differences to the results of the COMPASS survey described above could be attributed to different points in time in 2020 when the surveys were conducted. Furthermore, different survey methods were used: The COMPASS survey was conducted online, while the SOEP-CoV survey was conducted by telephone.

16 We also look at the change for individuals with children split by parental gender and education. Decreases in satisfaction with childcare and family life are roughly similar for mothers and fathers; however, overall, decreases in satisfaction with life are larger for women. Gaps by parental education in satisfaction with life overall and satisfaction with childcare that existed in the pre-Covid period appear to narrow slightly under Covid-19. There appears to be no differences in the level of satisfaction with family life in either period, despite the level shift downwards. Results are reported in the discussion paper version of this article, see Huebener et al. (2020).

17 Parents reporting a satisfaction level of zero are not included in the regressions, because log(0) is not defined.
